# Exploring the Impact of Age of Onset of Mild Cognitive Impairment on the Profile of Cognitive and Psychiatric Symptoms

**DOI:** 10.3390/geriatrics8050096

**Published:** 2023-09-25

**Authors:** Kleio Moustaka, Chrysanthi Nega, Ion N. Beratis

**Affiliations:** 1 Psychology Department, The American College of Greece, Deree, 6, Gravias Street, 153 42 Athens, Greece; k.moustaka.ps@gmail.com (K.M.); cnega@acg.edu (C.N.); 2 Alzheimer’s Center, “Nestor” Greek Psychogeriatric Association, 22, Ioannou Drosopoulou Street, 112 57 Athens, Greece; 3 1st Department of Neurology, Aiginiteio University Hospital, National and Kapodistrian University of Athens, 115 28, Athens, Greece

**Keywords:** MCI, neuropsychological evaluation, EOMCI, LOMCI, middle-onset MCI

## Abstract

The present study aims to explore the differences in the manifestation of cognitive decline and psychiatric symptoms across the different ages of MCI onset: early onset (EOMCI: <65 years old), middle onset (MOMCI: 65–75 years old), and late onset (LOMCI: >75 years old). It was hypothesized that individuals with EOMCI will preserve their cognitive functions to a greater extent as compared to individuals with LOMCI, even after adjusting the cognitive performance for age and education through the use of published Greek norms. The level of cognitive decline concerning MOMCI was evaluated for extracting more precise conclusions regarding the impact of the age of onset on the patterns of MCI symptomatology. The analyses of data were conducted in a Greek population of individuals with MCI, who were consecutive visitors of the Outpatient Memory Clinic of Nestor Alzheimer’s Centre in Athens, Greece. The sample consisted of 297 participants who fulfilled the following inclusion criteria: MCI diagnosis based on Petersen’s criteria, Greek mother language, and absence of a psychiatric history or chronic and incurable organic disease. The overall results support the presence of a cognitive advantage of the EOMCI group compared to the LOMCI group. In the MOMCI group, cognitive performance displayed a tendency to remain intermediate compared to the other two groups. Nonetheless, significant differences were observed when this group was compared with the LOMCI group. The current findings indicate that the age of onset should be taken under consideration in the neuropsychological assessment of individuals with MCI. The specific parameters could have implications in terms of prognosis as well as the design and implementation of tailored interventions.

## 1. Introduction

The rise of life expectancy observed recently in western countries has led to a significant increase in the elderly population and a concerning rise in the prevalence rates of dementia, especially of Alzheimer’s disease (AD). Despite conducting extensive research on the epidemiology, symptomatology, and etiology of dementia over the past thirty years, no effective treatment plan is yet available that can revert individuals to normal cognition (NC) in the vast majority of cases [1]. However, mounting evidence suggests that early diagnosis of cognitive decline, particularly in the Mild Cognitive Impairment (MCI) stage, may provide a promising ground for successful interventions and potential reversion to NC (e.g., [2,3,4]).

MCI is an intermediate clinical state in the course of a neurodegenerative disease (NDGD) that lies between normal cognitive aging and dementia. Individuals with MCI experience cognitive decline that is above the level of normal cognitive aging, while still maintaining appropriate levels of daily functionality, which means that they do not meet the criteria for a clinical diagnosis of dementia [5,6]. Individuals with MCI may fall into distinct categories based on the specific cognitive domain(s) in which they exhibit impairment. Petersen et al. (2009) [7] proposed two distinct categorical differentiations of MCI based on memory impairment and the number of affected cognitive domains. The first distinction categorizes individuals with amnestic MCI, i.e., with memory impairment, and non-amnestic MCI, where an absence of memory impairment is accompanied by a clinical deterioration in one or more memory-unrelated domains of cognition, such as executive functioning and visuospatial ability. The second differentiation refers to impairment in a single-domain MCI, as opposed to impairment in multiple-domain MCI, indicating a greater degree of brain neurodegeneration and consequently worse prognosis than single-domain MCI [8]. Thus, an individual with MCI may fall into one of four categories: non-amnestic single-domain MCI, non-amnestic multiple-domain MCI, amnestic single-domain MCI, or amnestic multiple-domain MCI.

All these distinctions contribute to the comprehension of the brain-breadth of the disease and its severity, prognosis, and the likelihood of progression to dementia. The early detection of MCI is crucial because individuals with MCI, even those who managed to revert to normal cognition at one point (20% reversion rate), have a higher and faster progression rate to dementia (10% to 15% per year), compared to people who never develop MCI [8]. The prevalence of MCI has been estimated to range from 5.3%, as stated by a Finnish study of a cohort of 806 individuals [9], to 7.7%, as reported in a systematic review of nine studies conducted in Europe, USA, and Brazil [10]. Mortality rates among individuals with MCI have been reported to be higher than cognitively healthy populations in Olmsted County, Minnesota, in the U.S. [11]. The incidence rates of MCI among older individuals in Sweden and in the U.S. (New York) also vary across both different subtypes and various age groups, with percentage rates that exceed 20% for individuals above 70 years old [12,13,14]. In a Greek population, the prevalence rate among older individuals has been found to be 5.4/100 person years [15].

In the course of MCI, individuals may experience deterioration in a variety of cognitive domains. One of the most prominent effect is episodic memory deterioration, which can be assessed for different modalities, namely verbal and non-verbal information by assessing the domains of immediate recall, delayed recall and recognition [16,17,18]. Visuospatial ability and executive functioning also play a key role in MCI, and can be evaluated through attention, psychomotor and processing speed, visual search speed and scanning, mental flexibility, ability to complete and adjust a plan, and the ability to maintain two trails of thought simultaneously [19,20]. Language impairment in areas including naming, fluency, comprehension, and expressive speech, may also be evident and can be measured through assessing both semantic (category) and phonemic (letter) fluencies. Overall, MCI is characterized by cognitive scores that vary from 1 to 1.5 standard deviations (SD) below the normative data based on age and level of education [8,21,22]. Nonetheless, individuals with MCI retain their autonomy and independence within normal levels despite the possibility of experiencing mild difficulties in the domain of functionality.

In addition to cognitive symptoms, individuals with MCI may also present various neuropsychiatric symptoms (NPSs), including anxiety, stress, depression, irritability, disinhibition, apathy, psychosis, delusions, hallucinations, aberrant motor behavior, psychomotor agitation, and euphoria [23,24,25,26]. High stress levels in people with MCI are often followed by sleep dysfunctions, such as insomnia, as well as compromised daily functionality [27,28].

While existing studies have differentiated MCI based on the extent of brain neurodegeneration and neuropsychiatric symptoms, there is limited literature on MCI and its distinct manifestation across the different ages of onset. Existing evidence suggests that age is a key factor in differentiating individuals with MCI [29]; however, research has mainly focused on the biological basis of age-related differentiation. For example, Tábuas-Pereira and colleagues (2016) [29] compared individuals with early-onset MCI (EOMCI) to people with late-onset MCI (LOMCI) on a number of cerebrospinal fluid (CSF) biomarkers as well as genetic and clinical background. Even though they found no differences in conversion rates to AD between the two groups, individuals with early-onset MCI exhibited better performance in brief cognitive tests compared to individuals with late-onset MCI. Some studies have focused on researching the prediction regarding the progression from MCI to dementia and/or AD [16,30,31], showing that the majority of cognitive tests are sensitive in differentiating the transition from MCI to dementia. Other studies have examined either neuropsychological/biological or possible sex differences between MCI and AD, with females exhibiting a more pronounced cognitive decline compared to males [32,33,34]. Furthermore, a study by Kim and colleagues (2010) [35] demonstrated that people with LOMCI had significantly poorer performance in verbal recall and word fluency compared to individuals with EOMCI. Additionally, Tábuas-Pereira et al. (2016) [29] found that individuals with LOMCI scored significantly lower than individuals with EOMCI on two brief cognitive tests: the Mini-Mental State Examination [36] and the Montreal Cognitive Assessment [37]. Although the aforementioned studies have provided useful information regarding MCI progression to dementia, prognosis, dementia-related biomarkers, and neuropsychological differences, the sample sizes have been quite limited, lacking a clear distinction of what constitutes EOMCI and LOMCI. Furthermore, classification has been mainly based on the general cut-off age of 65 for dementia [38], and the neuropsychological assessments utilized were not comprehensive enough to provide information about multiple significant cognitive functions.

The present study uses an extensive clinical and cognitive battery to assess MCI manifestation in three groups with a different age of onset: early (<65 years old), middle (65–75 years old), and late-onset (>75 years old) MCI. This approach aims to provide useful information for enhancing relevant knowledge regarding the impact of age on the patterns of cognitive symptomatology of individuals with MCI as well as to find possible connections between clinical measures (depression, anxiety, and stress) and the age of onset of the specific cognitive disorder.

The introduction of an additional MCI group, called middle-onset MCI (MOMCI), intends to provide additional information regarding the age-related neuropsychological trajectory of individuals that meet the specific clinical diagnosis. Due to scarcity of data on the differentiation of individuals with MCI based on their age of disease-onset, the present study aims to explore the manifestation of cognitive decline and psychiatric symptomatology across the different age-of-onset MCI groups (EOMCI, MOMCI, and LOMCI) in a Greek cohort of individuals with MCI. In order to control for the natural differences on cognition due to the ageing process, it was decided to use normative data that were standardized in terms of education and age, and not the initial raw scores on the various neuropsychological measures. To the best of our knowledge, this is a novel element of the current study because this specific approach has not been implemented by previous relevant research, and thus, it has the capacity to increase our insights regarding the true differences that could exist on the patterns of cognitive symptomatology among individuals with MCI with a different age of onset.

Based on existing findings from studies conducted in Korea and in Portugal, individuals with EOMCI show cognitive advantage compared to people with LOMCI [29,35]. Hence, it was hypothesized that the EOMCI group would perform better in all the assessed cognitive functions (including psychomotor speed and visual attention; verbal fluency, both semantic and phonemic; executive control; memory; immediate and delayed recall; recognition of a word-list) compared to the LOMCI group. This comparison will provide valuable insight into the cognitive differences between these groups since standardized scores of the aforementioned cognitive assessments will be used. This approach represents a unique aspect of the current study as participants’ z-scores were determined using established Greek normative data. Moreover, the level of cognitive functioning of the MOMCI group was evaluated according to its resemblance to either EOMCI or LOMCI on the aforementioned cognitive measures by implementing an exploratory perspective. Also, based on previous findings of the impact of gender and years of education on the cognitive functioning of older adults [39,40,41], the present study examined whether the specific factors have the capacity to moderate the impact of age of onset on the neuropsychological profile of MCI. Building upon existing literature that shows a different prevalence of MCI subtypes across different age groups of older people in Europe [13,42,43], it is expected that MCI subtype and MCI age-of-onset will be interrelated. More specifically, an increased frequency of multi-domain MCI is expected to be observed in the LOMCI group as compared to the EOMCI group. Lastly, we followed an exploratory approach without the development of a directional hypothesis for the differences on anxiety, depression, and stress between the three different age groups due to the absence of sufficient evidence according to the outcomes of previous relevant research [29].

## 2. Methods

### 2.1. Participants

This study included 297 patients with Mild Cognitive Impairment (MCI) (215 females; *M_age_* = 69.16, *SD_age_* = 9.16; *M_Education years_* = 11.80, *SD_education_* = 4.22;), categorized into three distinct groups: early-onset MCI [*n* = 89 (70 females); *M_age_* = 57.96, *SD_age_* = 5.07; *M_education_* = 13.25, *SD_education_* = 3.35], middle-onset MCI [*n* = 126 (93 females); *M_age_* = 70.37, *SD_age_* = 3.22; *M_education_* = 11.75, *SD_education_* = 4.27], and late-onset MCI [*n* = 82 (52 females); *M_age_* = 79.49, SD*_age_* = 3.82; *M_education_* = 10.32, *SD_education_* = 4.49] (Table 1). Participants in each group exhibited normal levels of daily functionality (see Table 2), and the MCI diagnosis was based on Petersen and Morris’ criteria. The years of education were significantly different (*F* (2, 294) = 11.01, *p* < 0.001) between all three age-of-onset groups: EOMCI (*M* = 13.25, *SD* = 3.35), MOMCI (*M* = 11.75, *SD* = 4.27), and LOMCI (*M* = 10.32, *SD* = 4.49). The sample comprised 15 patients with non-amnestic single-domain MCI, 23 patients with amnestic single-domain MCI, 36 patients with non-amnestic multiple-domain MCI, and 223 patients with amnestic multiple-domain MCI (see Table 3). The underlying pathological conditions with which the participants were diagnosed were the following: Alzheimer’s disease (*N_total_* = 199), cerebrovascular disease (*N_total_* = 52), or mixed pathology (*N_total_* = 46). For more information, see Table 4. In order to avoid selecting individuals with a highly divergent profile, it was decided not to include participants diagnosed with other pathological conditions, such as frontotemporal dementia, parkinsonian syndromes, Huntington’s’ disease and Lewy Body disease. Inclusion criteria were preserved daily functionality, Greek mother language, and absence of a psychiatric history or chronic and incurable organic disease. Also, according to the records and the information collected during the interview process from the participants and the family members, memory complaints or any other cognitive complaint of the participants should have a duration of up to one year before the conduction of the neuropsychological assessment. This strategy was implemented in the three different age-of-onset MCI groups to have a similar disease duration during the period that the data collection took place.

### 2.2. Materials

The diagnostic procedure of patients included the following valid and well-established instruments.

*Lawton–Brody Instrumental Activities of Daily Living* (*IADL*). The Lawton IADL 8-item scale assesses patients’ degree of autonomy in daily functioning [44]. An index of performance adequacy is calculated regarding the following eight daily functions: ability to use telephone, shopping, meal preparation, housekeeping, laundry, mode of transportation, responsibility for own medication, and financial management. Good levels of reliability were exhibited for this scale (Cronbach’s alpha: 0.809).

*Mini Mental State Examination* (*MMSE*). The MMSE is a measure of general cognitive status [45,46], evaluating attention, memory, language, time and space orientation, and visuospatial skills, with scores ranging from 0 (very severe cognitive impairment) to 30 (no cognitive impairment). Previous research has confirmed good levels of reliability regarding the MMSE scale (Cronbach’s alpha: 0.780) [47].

*REY Auditory Verbal Learning Test* (*RAVLT*). This test examines participants’ ability to encode, consolidate, store, and retrieve verbal information. It consists of a five-trial presentation of a 15-word list of nouns (list A), followed by a single presentation of a 15-noun interference list (list B), as well as two post-interference recall trials—one immediate and one delayed—and a final recognition trial comprising 50 words from lists A and B, and 20 distractor words semantically or phonemically similar to the words of the latter lists [48,49,50,51]. Statistical analysis showed high levels of reliability of the RAVLT scale (Cronbach’s alpha: 0.917).

*Verbal Fluency Test* (*VFT*). The VFT scale examines frontal and temporal lobes’ function and consists of two components, phonemic fluency (PF) and semantic fluency (SF). Similarly to Benton and Hamsher (1976) [52], who proposed the English letters “F”, “A”, and “S”, in PF, the examinee is asked to evoke words that begin with the Greek letters “Χ”, “Σ”, “A” within three minutes (one minute per letter) [53]. Similarly, in SF, the participant is required to evoke words that belong to the following categories—animals, fruits, and objects—within three minutes (one minute per category) [54]. Very good levels of reliability were represented by a Cronbach’s alpha of 0.873 for PF and 0.767 for SF.

*Trail Making Test A & B* (*TMT A & B*). The TMT test evaluates visual search speed, mental flexibility, executive functioning, and speed of processing, and consists of two parts, A and B [55]. In part A, the individual is required to connect numbers (1–13) in ascending order by drawing lines within 180 s. In part B, for a measure of executive control, the subject is asked to connect a series of numbers (1–13) and letters (A-L/A-Μ in the Greek version) in an ascending pattern, while alternating between numbers and letters (e.g., 1-A-2-B-3-C-4-D, etc.) within 300 s. In both parts, the individuals are instructed to complete the tasks as quickly as possible, without lifting the pencil or pen from the paper. Errors during the trail connection affect the final score of individuals.

*Depression Anxiety Stress Scale* (*DASS-21*). The DASS-21 evaluates the degree of psychological burden in three dimensions: depression, anxiety, and stress [56,57,58]. Specifically, the dimension of depression examines dysphoria, anhedonia, despair, self-depreciation, life depreciation, and lack of interest/participation; the dimension of anxiety evaluates arousal of the autonomous nervous system and its musculoskeletal effect, subjective experience of anxiety, and state anxiety; the stress dimension assesses temperament, restlessness, impatience, hyperarousal, and irritability/hyperreactivity. Each dimension has seven items, and scores range from 0 (none) to 3 (very much). Reliability analysis showed a good level of reliability for each DASS dimension: depression subscale (Cronbach’s alpha: 0.929), anxiety subscale (Cronbach’s alpha: 0.945), and stress subscale (Cronbach’s alpha: 0.923) [58,59].

### 2.3. Procedure

Participants of the present study were consecutive visitors of the Outpatient Memory Clinic of Nestor Alzheimer’s Centre, meeting the following inclusion criteria: complaints about cognitive disfunction, absence of a psychiatric history, Greek mother language, and a Mild Cognitive Impairment (MCI) diagnosis based on Petersen’s criteria [6]. All participants completed a thorough neuropsychological and medical evaluation after providing a written consent. The assessment, which lasted approximately 120 min, was conducted by professional psychologists, neuropsychologists, and psychiatrists, and comprised several sociodemographic questions, a brief medical history, and the administration of Lawton–Brody IADL, MMSE, RAVLT, VFT (both PF and SF), Trail Making Test (TMT, both parts A and B), and DASS-21. Finally, the Greek standardized version of each scale was used for the data collection process.

### 2.4. Ethical Considerations

The study was approved by both institutions involved in the research, (a) the Psychology Ethics Research Committee, operating under the auspices of the Institutional Review Board at the American College of Greece and (b) the Ethics Committee of the Alzheimer’s Outpatient Day Center, “Nestor” Greek Psychogeriatric Association. Informed consent was obtained from all individuals that took part; it was explained to them that participation was on a voluntary basis and that they had the right to withdraw at any time. Participants were informed about the nature of the study, the duration of their engagement, and the type of information that they would be asked to provide during the data collection process. Furthermore, participants were notified about the confidentiality of the procedure, and that the use of their background information would be only for research purposes. Participation was voluntary, and no compensation was offered.

## 3. Statistical Analyses

Statistical analyses of data were performed after converting raw scores into z-standardized scores, concerning the following cognitive tests: RAVLT (trials A1, A5, A7, and A8), verbal fluencies (both semantic and phonemic), and TMT (both trails A and B). Z-standardization of raw scores was based on Greek norms of cognitively healthy populations [53,60,61]. Furthermore, the TMT z-scores were inverted through multiplying by −1. Finally, a grand Z-score index that took the following cognitive measures under consideration was developed: RAVLT A5, RAVLT A7, RAVLT A8, VFT SF, VFT PF, TMT A, and TMT B. The statistical significance was set at the level of 0.05, and in the case of post hoc comparisons for statistically significant ANOVA models, the Bonferroni correction was applied.

*Reliability Analyses.* Reliability analyses were utilized to calculate the Cronbach’s alpha values of the following measures based on raw scores: IADL (all 8 items), RAVLT (A1-A8), VFT (SF and PF, separately), and AIS-8 (all 8 items).

*Analyses of Variance and Covariance* (*ANOVA and ANCOVA*). One-way ANOVA analyses were utilized to measure the effects of age of MCI onset (EOMCI: <65 years old, MOMCI: 65–75 years old, and LOMCI: >75 years old) on the levels of cognition, as well as on depression, anxiety, and stress. Two-way ANOVA analyses were used to measure the effects of age of MCI onset and gender, as well as of education (Group 1: 1–9 years, Group 2: 9–12 years, Group 3: >12 years), on the levels of cognition. ANCOVA analyses were used to measure the effects of age of MCI onset on the levels of cognition, after controlling for the effect of the MMSE score and education.

*Chi-Square for Independence.* A chi-square of independence test was conducted to examine the association between age of onset and type of MCI, namely amnestic MCI single-domain, amnestic MCI multiple-domain, non-amnestic MCI single-domain, and non-amnestic MCI multiple-domain. In addition, the MCI type (single-domain/multiple-domain) was treated as a binary variable in order to further explore its association with the age of onset.

## 4. Results

### 4.1. Patterns of Cognition in Groups of Patients with EOMCI, MOMCI, and LOMCI

One-way ANOVA analyses revealed statistically significant effects of the MCI age-of-onset (EOMCI, MOMCI, and LOMCI) on the following cognitive measures: RAVLT A1 (*F*(2, 294) = 5.039, *p* = 0.033), RAVLT A7 (2, 291) = 6.304, *p* = 0.002), RAVLT A8 (*F*(2, 288) = 6.999, *p* = 0.001), VFT SF (*F*(2, 294) = 16.028, *p* < 0.001), VFT PF (*F*(2, 293) = 3.681, *p* = 0.026, TMT A (*F*(2, 291) = 14.878, *p* < 0.001), TMT B (*F*(2, 260) = 7.979, *p* < 0.001), and the grand z-score index (*F*(2, 260) = 13.711, *p* < 0.001). However, no statistically significant results were found for RAVLT A5 (*F*(2, 292) = 0.631, *p* = 0.533). Further analysis using the Bonferroni correction for post hoc comparisons revealed specific differences among the MCI groups. MOMCI showed statistically significant differences from LOMCI in RAVLT A1, while EOMCI significantly differed from LOMCI in both RAVLT A7 and RAVLT A8. Additionally, all groups exhibited differences from one another regarding VFT SF, while in VFT PF there was only a difference between EOMCI and LOMCI. Analyses of both TMT A and TMT B indicated that EOMCI significantly differed from LOMCI, and MOMCI differed from LOMCI. For more information, see Table 5.

To account for the influence of general cognitive status, as measured via MMSE, a series of ANCOVA models were conducted. After controlling for the effect of MMSE as an indicator of general cognitive status, a consistent pattern of results emerged, aligning with previous analyses. Specifically, the analyses revealed statistically significant effects of the MCI age-of-onset (EOMCI, MOMCI, and LOMCI) on the following cognitive measures, even after controlling for the effect of MMSE: RAVLT A1 (*F*(2, 290) = 4.443, *p* = 0.013, *partial η*^2^ = 0.030), RAVLT A7 (*F*(2, 287) = 3.188, *p* = 0.043, *partial η*^2^ = 0.022), RAVLT A8 (*F*(2, 284) = 3.242, *p* = 0.041, *partial η*^2^ = 0.022), VFT SF (*F*(2, 290) = 8.335, *p* < 0.001, *partial η*^2^ = 0.054), TMT A (*F*(2, 287) = 7.228, *p* = 0.001, *partial η*^2^ = 0.048), and TMT B (*F*(2, 256) = 4.228, *p* = 0.016, *partial η*^2^ = 0.032). However, no statistically significant results were found for RAVLT A5 (*F*(2, 288) = 3.803, *p* = 0.084, *partial η*^2^ = 0.017), and VFT PF (*F*(2, 289) = 0.839, *p* = 0.433, *partial η*^2^ = 0.006). For more information, see Table 6.

Similarly, in order to control for the role of education, a series of ANCOVA models was conducted as well. The pattern of findings that was obtained was in line with the initial ANOVA models. More specifically, after controlling for the role of education, the “MCI age of onset” retained its statistical significance in all cases that were significant according to the initial ANOVA models: RAVLT A1 (*F*(2, 293) = 3.316, *p* = 0.038), RAVLT A7 (2, 290) = 7.495, *p* = 0.001), RAVLT A8 (*F*(2, 287) = 7.323, *p* = 0.001), VFT SF (*F*(2, 293) = 24.303, *p* < 0.001), VFT PF (*F*(2, 292) = 5.669, *p* = 0.004, TMT A (*F*(2, 290) = 11.236, *p* < 0.001), and TMT B (*F*(2, 259) = 5.398, *p* = 0.005). In addition, education, as a covariate, was significantly associated with the following cognitive measures: VFT SF (*F*(1, 293) = 21.228, *p* < 0.001), VFT PF (*F*(1, 292) = 7.877, *p* = 0.005, TMT A (*F*(1, 290) = 8.229, *p* = 0.004), and TMT B (*F*(1, 259) = 30.352, *p* < 0.001).

Also, in order to provide further support to the main body of the analysis, a correlation analysis was applied between the age of onset of the participants and their standardized scores on the cognitive measures that revealed significant negative associations in the majority of the corresponding cases: TMT A (r = −0.310, *p* < 0.001), TMT B (r = −0.270, *p* < 0.001), VFT SF (r = −0.331, *p* < 0.001), VFT PF (r = −0.151, *p* = 0.009), RAVLT A7 (r = −0.230, *p* < 0.001), RAVLT A8 (r = −0.262, *p* < 0.001).

### 4.2. Interaction Effects of MCI Age-of-Onset and Gender on Cognition

Two-way ANOVA analyses showed no statistically significant interaction effects of gender and MCI age-of-onset on the following cognitive measures: RAVLT A1 (*F*(2, 291) = 0.436, *p* = 0.647, *partial η*^2^ = 0.003), RAVLT A5 (*F*(2, 289) = 0.043, *p* = 0.958, *partial η*^2^ < 0.001), RAVLT A7 (*F*(2, 288) = 1.711, *p* = 0.183, *partial η*^2^ = 0.012), RAVLT A8 (*F*(2, 285) = 0.765, *p* = 0.466, *partial η*^2^ = 0.005), VFT SF (*F*(2, 291) = 1.257, *p* = 0.286, *partial η*^2^ = 0.009), VFT PF (*F*(2, 290) = 0.270, *p* = 0.764, *partial η*^2^ = 0.002), TMT A (*F*(2, 288) = 0.967, *p* = 0.382, *partial η*^2^ = 0.007), and TMT B (*F*(2, 257) = 0.550, *p* = 0.577, *partial η^2^* = 0.004). For more information, see Table 7.

### 4.3. Levels of Depression, Anxiety, and Stress across the Different MCI-Onset Groups

One-way ANOVA analyses revealed no statistically significant differences between EOMCI, MOMCI, and LOMCI regarding their levels of DASS depression (*F*(2, 294) = 0.846, *p* = 0.430, *partial η*^2^ = 0.008), anxiety (*F*(2, 294) = 0.248, *p* = 0.781, *partial η*^2^ = 0.002), and stress (*F*(2, 294) = 0.287, *p* = 0.751, *partial η*^2^ = 0.003; see Table 8).

### 4.4. Prevalence of MCI Types across the Three Age-of-Onset Groups

**Figure 1:**
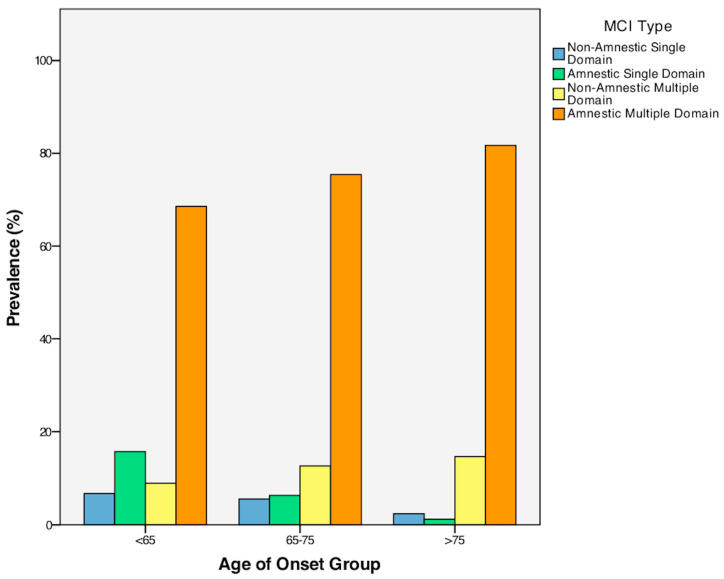
Prevalence of MCI types across the three age groups.

A Chi-square test for independence revealed a statistically significant association between MCI type (amnestic single-domain MCI, amnestic multiple-domain MCI, non-amnestic single-domain MCI, and non-amnestic multiple-domain MCI) and the age of onset of this clinical condition, *χ*^2^ (6, *n* = 297) = 16.00, *p* = 0.014, *phi* = 0.232 (see Figure 1). Specifically, this association indicates the reduced frequency of single-domain amnestic and non-amnestic MCI in older adults compared to EOMCI and MOMCI. Notably, within the overall sample, only 5.1% was diagnosed with non-amnestic single-domain MCI, 7.7% with amnestic single-domain MCI, and 12.1% with non-amnestic multiple-domain MCI, while the majority of the participants (75.1%) belonged to the amnestic multiple-domain MCI category. For more information, see Table 3 above. In addition, a Chi-square test analysis was conducted for exploring the association between MCI types, treated as a binary variable (multiple-domain/single-domain), and age-of-onset MCI groups (EOMCI/MOMCI/LOMCI). According to the obtained results, the overall model was significant, χ^2^ (2, Ν = 297) = 13.69, *p* < 0.001. More specifically, the analysis revealed that the multiple-domain MCI was met more commonly in the case of the MOMCI group (χ^2^ (1, Ν = 215) = 4.27), *p* = 0.039) and the LOMCI group (χ^2^ (1, Ν = 171) = 12.98, *p* < 0.001), as compared to the EOMCI group. Also, the multiple-domain MCI was met more commonly in the case of the LOMCI group (χ^2^ (1, Ν = 208) = 4.27), *p* = 0.039), as compared to MOMCI.

## 5. Discussion

The aim of the present study was to investigate the distinct elements of the cognitive and psychiatric profile of individuals with EOMCI, MOMCI and LOMCI. In the case of the cognitive measures, standardized scores were used based on norms especially developed for the Greek population. Overall, the findings supported the main hypothesis, as individuals with EOMCI exhibited better cognitive performance than LOMCI individuals across all examined cognitive domains. This comparison provides useful information regarding the genuine differences that exist in terms of the cognitive symptoms between the different age-of-onset groups because the standardized scores that were utilized were adjusted for age and education according to published Greek norms. In addition, the specific pattern of performance was retained even after treating the years of education and MMSE performance as covariates in the analysis in order to control for the role of education and general cognitive status, respectively. Along this vein, a complementary result that supports the direction of the main findings is that despite the adjustment that was carried out to the data, a negative association was observed between the age of onset of MCI and performance on the majority of the cognitive measures. Regarding the MOMCI group, cognitive performance had a tendency to remain intermediate as compared to the other two groups. Nonetheless, a greater number of post hoc comparisons reached the level of statistical significance when the MOMCI group was compared with the LOMCI group. On the other hand, non-significant differences were observed in the case of the levels of anxiety, depression and stress between the three different age-of-onset MCI groups. Hence, this null finding indicates that the present observations regarding the cognitive profile of individuals with MCI were not influenced by differences on their psychiatric state according to the age of onset.

Specifically, individuals with EOMCI demonstrated significantly greater performance in both delayed recall (RAVLT A7) and recognition (RAVLT A8) compared to individuals with LOMCI. However, there were no significant differences in immediate recall (RAVLT A1 and A5). Furthermore, EOMCI individuals performed significantly better in verbal ability and speed of processing (both SF and PF of the VFT) compared to individuals with LOMCI. According to the performance on part-A and part-B of the TMT, individuals with LOMCI showed significantly lower scores in visuomotor coordination, speed of visual attention and aspects of executive functioning, namely mental flexibility and inhibitory control, compared to individuals with EOMCI.

Additionally, this study found a higher prevalence of amnestic single-domain pathology among individuals with EOMCI compared to LOMCI and MOMCI. In contrast, a higher number of individuals with LOMCI exhibited amnestic multiple-domain pathology compared to individuals with EOMCI. In addition, the multiple-domain MCI in general was met more commonly in the case of the MOMCI group and the LOMCI group, as compared to the EOMCI group. Also, the multiple-domain MCI was met more commonly in the case of the LOMCI group, as compared to MOMCI. This pattern of results may indicate that individuals with LOMCI exhibit a more extensive spread of the underlying pathology compared to EOMCI individuals; this is in line with the findings of the main body of the analysis that revealed accentuated cognitive symptoms in the older “age of onset” groups. Such a differentiation between individuals with EOMCI and LOMCI could have important implications when considering the type of cognitive training that those patients will need, the pattern and degree of difficulty that may be observed in complex activities of daily living such as driving, and the level of risk regarding the progression to dementia. Along this line, Cheng et al. (2012) [62] pointed out that single-domain cognitive training enhanced constructional/visuospatial and attention capacities, whereas multiple-domain cognitive training improved memory proficiency. Similarly, Whitwell et al. (2007) [63] explored the distinction between single-domain and multiple-domain spread of pathology in the brain. Specifically, their findings showed that although both amnestic single-domain MCI and amnestic multiple-domain MCI exhibited medial and inferior-temporal-lobes loss, the amnestic multiple-domain MCI group also presented additional impairment in brain regions such as the posterior cingulate, parietal association cortex, and posterior temporal lobe. Furthermore, this previous study demonstrated that non-amnestic single-domain MCI individuals with language deficits showed brain atrophy in the left anterior inferior temporal lobe, while those with attention/executive impairment exhibited atrophy in the hypothalamus and the basal forebrain.

Younger individuals with MCI have been found to display faster reaction times to previously learned items [64], indicating enhanced activity in the regions of posterior cingulate, lateral and medial parietal lobes [31]. These earlier findings appear to support the moderating role of age in terms of the cognitive and functional patterns of individuals with MCI, which might also provide a base for explaining the superior cognitive performance of individuals with EOMCI compared to those with LOMCI that was observed in the current study. It is also possible that when MCI pathology manifests at an earlier age, the overall structural and functional integrity of the brain is preserved to a greater extent, thus leading to a smaller degree of cognitive attenuation compared with late-onset individuals. Several previous studies suggest that even cognitively healthy older individuals show signs of cognitive decline, especially in episodic memory, visuospatial ability, speeded motor ability, and confrontation naming, and that this pattern of performance accentuates with the advancement of age [42,65,66,67,68,69]. Nonetheless, this does not suggest that younger adults with MCI automatically have a better prognosis. As the current literature suggests, MCI in younger populations appears to be heterogeneous regarding long-term prognosis [70]. Possibly, in some cases, a younger age-of-onset may imply a more aggressive underline pathology, thus leading to a faster progression of cognitive and clinical symptomatology [71,72]. Hence, future research is required in this direction in order to enhance the existing knowledge.

In the current discussion, it is important to consider the vascular changes that occur in the human brain as a result of ageing which gradually accentuate due to this process. MCI typically serves as a prodromal stage for dementia, including vascular dementia or AD. Specifically, the amnestic types of MCI are associated with a high risk of progression to AD [73,74], which, in turn, has been related to prior vascular impairments [75]. There is accumulating evidence connecting age-relevant structural alterations and dysfunction of capillaries, cerebral arteries to the pathogenesis of various dementia types, as well as AD [76,77]. These pathologies, that may be observed in patients at risk of developing or suffering from AD [78,79,80], show a positive association with the factor of age [81,82,83,84]. Additionally, recent research has highlighted the role of damage in strategic white matter tracts, secondary neurodegeneration, microinfarcts, loss of microstructural tissue integrity, and microhemorrhages as a part of the ageing process that correlates with cognitive decline [85]. Hence, according to the previously presented information, the older group of patients with MCI appear to have a more vulnerable neural substrate that may explain, at least partially, the augmented cognitive symptoms that were observed in the specific clinical group.

The present study also explored the influence of education on the cognitive measures of the study through the use of a set of ANCOVA models. According to this analysis, the age-of-onset retained its statistical significance on the cognitive profile of individuals with MCI in all cases that were significant according to the initial ANOVA models. Nonetheless, a significant positive association of education was observed with the following cognitive measures: TMT A, TMT B, VFT PF, and VFT SF. Hence, these findings support the protective role of education against cognitive decline, just like the previous studies which have also indicated this according to the theory of cognitive reserve [86,87,88,89,90,91]. Notably, the specific protective mechanism appears to have the capacity to retain its role above and beyond the standardization process that was implemented on the data, through the use of published Greek norms that are adjusted for age and education.

Additionally, the differences in cognitive performance between individuals with EOMCI and individuals with LOMCI that were in line with our hypothesis, and thus, the present study supports the importance of including an intermediate group, namely individuals with MOMCI, when exploring the cognitive profile of MCI according to the age of onset of this clinical condition. The MOMCI group is a newly introduced categorization that aims to further explore the distinct impact of MCI age-of-onset on the cognitive and psychiatric profile of this clinical group. Aging is a critical factor that affects individuals who are either cognitively healthy or within the MCI spectrum, and therefore, its thorough evaluation appears to be advantageous. Specifically, MOMCI individuals demonstrated non-significant differences compared to EOMCI patients across most examined cognitive functions, including phonemic fluency, delayed recall, recognition, and executive functioning. However, a significant difference was observed between the two groups on the measure of SF. Between the MOMCI and LOMCI groups, significant differences were reported in the cases of immediate recall, semantic fluency, as well as for both TMT A and B. Importantly, when integrating the performance across the various cognitive domains through the use of a grand Z-score index, the differences between all the groups surpassed the level of statistical significance. Hence, this last finding clearly supports MOMCI’s tendency to remain intermediate, regarding their cognitive performance, compared to the other two groups.

The aforementioned results, together with the significant differences that were observed in the majority of cognitive measures between EOMCI and LOMCI, indicate that the MOMCI group displayed an overall tendency to remain intermediate, in terms of cognitive functioning, as compared to the other two groups. This can be attributed to the fact that ageing initially affects certain brain regions related to specific cognitive functions more than others, and the fact that the process of cognitive decline has a gradual nature. Indicative cognitive domains appearing to gradually decline as a function of age that could be related to the pattern of findings that was observed in the current study are the following: memory, executive control, attention-related functions and generally higher-order cognitive functions [92,93,94,95,96]. Along this line, previous research suggests that white matter in frontal regions is more sensitive to the ageing process [82], and that alterations in brain connectivity play a significant role in age-related decline in executive functioning [97,98]. Lastly, cohort effects could also contribute to the different patterns of cognitive performance across the three different MCI groups. In this direction, generational differences, including environmental exposures, educational experiences, and lifestyle patterns, may have the cognitive profile of the participants to an extent [68].

Though this study has provided valuable insights into the neuropsychological aspects of MCI manifestation across the age-of-onset spectrum, it has some limitations. Firstly, although according to the records and the information collected during the interview process, the overall sample had memory complaints or any other cognitive complaint up to one year before the conduction of the neuropsychological assessment, the exact onset of the MCI condition for the participants of this study is very difficult to be specified. Nonetheless, effort was undertaken to carefully develop the inclusion criteria of the study to control this important parameter to a great extent. Secondly, despite having a well-balanced sample in terms of the number of patients in each age-of-onset group, as well as their levels of education, psychiatric symptoms, and their medical background, it would have been more helpful to collect a more balanced sample based on gender. This would have allowed for a more thorough exploration of potential gender differences between EOMCI, MOMCI, and LOMCI in cognitive performance which could have provided further valuable insights. Hence, this specific research goal could serve as a reasonable target for future studies in this area. Also, this study was carried out in a single center and not in multiple centers, and therefore, prospective multicenter research could enhance the external validity of the current findings. Finally, the current findings may be enriched by exploring the role of genetic factors, lifestyle variables and the pathological condition underlying the cause for the development of MCI.

Although there is significant clinical and etiological heterogeneity in characterizing MCI, the unifying greater risk of progressing to dementia is evident. Recent research have focused on developing a standardized diagnostic classification for MCI that reflects the evolving knowledge in the field as well as on the development of effective non-pharmaceutical interventions. In this direction, the findings of the current study highlight the importance of the factor of age on the profile and extent of the cognitive symptoms that may be observed within the clinical spectrum of MCI. Future studies should target the exact brain functions that seem to decline in a quicker manner with ageing, aiming to create specialized cognitive strengthening programs that focus on these functions according to the group’s age. There is mounting evidence showing that diagnosis of MCI at an early stage, along with quick plans of action that include the implementation of tailored interventions, may either provide a better prognosis regarding progression to dementia by increasing the duration of MCI state, or even act protectively against dementia and revert to normal cognition [99,100,101].

To conclude, the increasing life expectancy of older people calls for vital actions regarding their medical and mental health, but first and foremost, their cognitive health. In this direction, the detailed evaluation of the cognitive profile of individuals with MCI that takes under consideration the factor of age of onset may facilitate the diagnostic and prognostic accuracy, as well as pave the way for the implementation of tailored interventions that accounts for the unique needs and characteristics of each member of this vulnerable group of our society.

## Figures and Tables

**Table 1:** Sociodemographic characteristics and brief medical history of participants (N = 297).

Participants’ Characteristics	Number of Participants	Statistics
	N (%)	Mean (SD)
Overall Sample		
Age		69.16 (9.16)
Education		11.80 (4.22)
MMSE		27.39 (2.37)
IADL		8.25 (2.58)
Gender		
Female	215 (72.4)	
Male	82 (27.6)	
Occupational Status		
Currently Working	59 (20.3)	
Unemployed	9 (3.1)	
Pensioner/Home Economics	222 (76.6)	
Handedness		
Right-handed	203 (94.4)	
Left-handed	8 (3.7)	
Ambidextrous	4 (1.9)	
Family Status		
Single	14 (4.7)	
Partnership	2 (0.7)	
Married	170 (57.4)	
Divorced	45 (15.2)	
Widowed	65 (21.9)	
Medical History		
Elevated Blood Pressure	125 (42.5)	
Diabetes	48 (16.7)	
Hypercholesterolemia	142 (49.8)	
Stroke	13 (4.6)	
Heart Attack	11 (3.9)	
Traumatic Brain Injury	29 (10.2)	
Smoking	66 (23.0)	
Physical Exercise	52 (18.1)	
Dementia Medication	15 (5.2)	
Psychiatric Medication	110 (38.2)	
General Medication	235 (82.2)	
Dementia Family History	100 (35.5)	
Cardiovascular Disease Family History	122 (43.4)	

**Table 2:** Descriptive statistics: levels of daily functionality across gender and age-of-onset groups.

Lawton IADL	Mean (SD)	N (%)
Age Group		
EOMCI	7.77 (0.14)	89 (30.0)
MOMCI	8.14 (0.19)	126 (42.4)
LOMCI	8.93 (0.22)	82 (27.6)
Gender		
Females	8.78 (0.17)	215 (72.4)
Males	6.86 (0.27)	82 (27.6)

**Table 3:** MCI categorization and MCI age-of-onset (N = 297).

Participants’ Categorizations	Number of Participants	Statistics
	N (%)	Mean Age (SD)
EOMCI (<65 years old)	89 (29.7)	57.96 (5.07)
MCI Categorization 1		
Non-Amnestic	14 (15.7)	
Amnestic	75 (84.3)	
MCI Categorization 2		
Single-Domain	20 (22.5)	
Multiple-Domain	69 (77.5)	
MCI Overall Categorization		
Non-Amnestic Single-Domain	6 (6.7)	
Non-Amnestic Multiple-Domain	8 (9.0)	
Amnestic Single-Domain	14 (15.7)	
Amnestic Multiple-Domain	61 (68.5)	
MOMCI (65–75 years old)	126 (42.4)	70.37 (3.22)
MCI Categorization 1		
Non-Amnestic	23 (18.3)	
Amnestic	103 (81.7)	
MCI Categorization 2		
Single-Domain	15 (11.9)	
Multiple-Domain	111 (88.1)	
MCI Overall Categorization		
Non-Amnestic Single-Domain	7 (5.6)	
Non-Amnestic Multiple-Domain	16 (12.7)	
Amnestic Single-Domain	8 (6.3)	
Amnestic Multiple-Domain	95 (75.4)	
LOMCI (>75 years old)	82 (27.6)	79.49 (3.82)
MCI Categorization 1		
Non-Amnestic	14 (17.1)	
Amnestic	68 (82.9)	
MCI Categorization 2		
Single-Domain	3 (3.7)	
Multiple-Domain	79 (96.3)	
MCI Overall Categorization		
Non-Amnestic Single-Domain	2 (2.4)	
Non-Amnestic Multiple-Domain	12 (14.6)	
Amnestic Single-Domain	1 (1.2)	
Amnestic Multiple-Domain	67 (81.7)	

**Table 4:** MCI underlying pathology categorization and MCI age-of-onset (N = 297).

Participants’ Categorizations	Number of Participants	Statistics
	N (%)	Mean Age (SD)
EOMCI (<65 years old)	89 (29.7)	57.96 (5.07)
AD	64 (71.9)	
CVD	15 (16.9)	
MP	10 (11.2)	
MOMCI (65–75 years old)	126 (42.4)	70.37 (3.22)
AD	84 (66.7)	
CVD	21 (16.7)	
MP	21 (16.7)	
LOMCI (>75 years old)	82 (27.6)	79.49 (3.82)
AD	51 (62.2)	
CVD	16 (19.5)	
MP	15 (18.3)	

Note. AD = Alzheimer’s disease, CVD = cerebrovascular disease, MP = mixed pathology.

**Table 5:** Cognitive comparisons among the three age groups (EOMCI, MOMCI, and LOMCI).

			EOMCI	MOMCI	LOMCI
Cognition	Age Group	Mean (SD)	Sig.		
RAVLT A1	EOMCI	−0.0129 (0.9739)	-	0.631	0.595
	MOMCI	−0.2231 (0.9251)	0.631	-	0.028 *
	LOMCI	0.2257 (1.7194)	0.595	0.028 *	-
RAVLT A5	EOMCI	0.1396 (1.1510)	-	1.000	1.000
	MOMCI	0.0241 (1.2634)	1.000	-	0.816
	LOMCI	0.2246 (1.4281)	1.000	0.816	-
RAVLT A7	EOMCI	0.2364 (1.3089)	-	0.103	0.001 *
	MOMCI	−0.2018 (1.4293)	0.103	-	0.245
	LOMCI	−0.5714 (1.7217)	0.001 *	0.245	-
RAVLT A8	EOMCI	−0.7474 (1.2914)	-	0.162	0.001 *
	MOMCI	−1.1872 (1.5977)	0.162	-	0.093
	LOMCI	−1.6942 (1.6558)	0.001 *	0.093	-
VFT SF	EOMCI	−0.2000 (1.2437)	-	0.007*	<0.001 *
	MOMCI	−0.6782 (1.0711)	0.007 *	-	0.007 *
	LOMCI	−1.1677 (1.0370)	<0.001 *	0.007*	-
VFT PF	EOMCI	−0.4347 (1.093)	-	0.187	0.025 *
	MOMCI	−0.7208 (1.1347)	0.187	-	0.890
	LOMCI	−0.8841 (1.0543)	0.025 *	1.000	-
TMT A	EOMCI	0.3597 (1.0499)	-	0.054	<0.001 *
	MOMCI	−0.2122 (1.4561)	0.054	-	0.001 *
	LOMCI	−1.0837 (2.5266)	<0.001 *	0.001*	-
TMT B	EOMCI	−0.3558 (2.8603)	-	0.270	<0.001 *
	MOMCI	−1.0335 (2.7005)	0.270	-	0.027 *
	LOMCI	−2.1846 (2.8602)	<0.001 *	0.027*	-

Note. Four decimals are kept with the aim of accurately describing z-standardized scores. The statistical significance was set at the level of 0.05, after controlling for the Bonferroni correction. * significant comparisons.

**Table 6:** MMSE scores comparisons among the three age groups (EOMCI, MOMCI, and LOMCI).

			EOMCI	MOMCI	LOMCI
	Age Group	Mean (SD)	Sig.		
MMSE	EOMCI	28.34 (1.71)	-	0.011	<0.001
	MOMCI	27.41 (2.32)	0.011	-	0.004
	LOMCI	26.37 (2.64)	<0.001	0.004	-

Note. The statistical significance was set at the level of 0.05, after controlling for the Bonferroni correction.

**Table 7:** Descriptive statistics of interaction effects of gender and MCI age-of-onset on cognition.

	EOMCI		MOMCI		LOMCI	
	Females	Males	Females	Males	Females	Males
Cognition	M (SD)	M (SD)	M (SD)	M (SD)	M (SD)	M (SD)
RAVLT A1	−0.1875 (0.9766)	0.0088 (0.9902)	−0.1340 (0.9305)	−0.4744 (0.8748)	0.3139 (1.6904)	0.0730 (1.7872)
RAVLT A5	0.1975 (1.1300)	−0.0708 (1.2339)	0.1136 (1.2688)	−0.2281 (1.2321)	0.3721 (1.5093)	−0.0263 (1.2606)
RAVLT A7	0.3223 (1.2980)	−0.0755 (1.3360)	−0.0888 (1.5041)	−0.5308 (1.1437)	−0.1354 (1.7608)	−1.3125 (1.3891)
RAVLT A8	−0.5919 (1.2122)	−1.3039 (1.4423)	−1.1097 (1.5695)	−1.4127 (1.6822)	−1.3627 (1.8835)	−2.2656 (2.0269)
VFT SF	−0.2246 (1.3081)	−0.1094 (0.9959)	−0.6021 (1.0775)	−0.8928 (1.0389)	−0.9893 (0.9689)	−1.4769 (1.0940)
VFT PF	−0.3997 (1.1772)	−0.5619 (0.7212)	−0.6094 (1.1512)	−1.0349 (1.0400)	−0.7564 (0.9471)	−1.1054 (1.2030)
TMT A	−0.3071 (1.0474)	−0.3356 (0.6819)	−0.6057 (0.9546)	−0.9638 (0.8779)	−0.8728 (0.81929)	−1.2912 (1.0015)
TMT B	0.3941 (1.0609)	0.2348 (1.0272)	−0.1595 (1.4550)	−0.3652 (1.4718)	−1.2641 (2.8748)	−0.7771 (1.7906)

Note. Four decimals are kept with the aim of accurately describing z-standardized scores.

**Table 8:** Descriptive statistics: level of DASS depression, anxiety and stress across the different MCI onset groups.

Age Group	Depression	Anxiety	Stress
EOMCI	4.90 (4.93)	2.87 (3.47)	5.57 (4.52)
MOMCI	4.23 (4.70)	3.25 (3.74)	5.18 (4.54)
LOMCI	5.27 (4.77)	3.05 (3.41)	4.95 (4.39)

Note. The presented descriptive statistics are mean (standard deviation).

## Data Availability

For data availability and access, please contact: k.moustaka.ps@gmail.com.
